# Exploration of isoxanthohumol bioconversion from spent hops into 8-prenylnaringenin using resting cells of *Eubacterium limosum*

**DOI:** 10.1186/s13568-020-01015-5

**Published:** 2020-04-24

**Authors:** Esther Moens, Selin Bolca, Tom Van de Wiele, Anita Van Landschoot, Jan L. Goeman, Sam Possemiers, Willy Verstraete

**Affiliations:** 1 ProDigest BVBA, Technol Pk 82, 9052 Ghent, Belgium; 2 Ugent, CMET, Coupure Links 653, 9000 Ghent, Belgium; 3 Ugent, Dept Biotechnology, Valentin Vaerwyckweg 1, 9000 Ghent, Belgium; 4 Ugent, Dept Organic and Macromolecular Chemistry, Krijgslaan 281-S4, 9000 Ghent, Belgium; 5 AVECOM NV, Ind Weg 122P, 9032 Wondelgem, Belgium

**Keywords:** *Eubacterium limosum*, Hop fermentation, Antibacterial, Isoxanthohumol, 8-prenylnaringenin, Industrial fermentation

## Abstract

Hops is an almost unique source of the potent phytoestrogen 8-prenylnaringenin (8-PN). As hops contain only low levels of 8-PN, synthesis may be more attractive than extraction. A strain of the Gram-positive *Eubacterium limosum* was isolated previously for 8-PN production from more abundant precursor isoxanthohumol (IX) from hops. In this study, spent hops, an industrial side stream from the beer industry, was identified as interesting source of IX. Yet, hop-derived compounds are well-known antibacterial agents and the traces of a large variety of different compounds in spent hops interfered with growth and IX conversion. Critical factors to finally enable bacterial 8-PN production from spent hops, using a food and feed grade medium, were evaluated in this research. The use of bacterial resting cells and complex medium at a pH of 7.8–8 best fulfilled the requirements for 8-PN production and generated a solid basis for development of an economic process.

## Introduction

The prenylated flavanone 8-prenylnaringenin (8-PN) is known as a very potent phytoestrogen (Milligan et al. 1999; Schick and Schwack 2018) and is almost exclusively found in hops. Yet, concentrations of 8-PN are only 0.025–0.060 g kg^−1^ in hop strobili (often called hop cones) (Rong et al. 2000). This fact hampers commercially viable recovery of 8-PN through extraction and makes (bio-) synthesis more attractive to obtain the compound at large quantities. Xanthohumol (X) is a primary chalcone in hops, present at 0.1–1% on dry weight in the lupulin glands (Stevens et al. 1999a). Isoxanthohumol (IX) is readily formed from X by acid‐catalyzed cyclization in the acidic conditions of the upper gastrointestinal tract (Nikolic et al. 2005), by chemical isomerization in alkaline conditions (Stevens et al. 1999b; Wilhelm and Wessjohann 2006; Kamiński et al. 2017), or by enzymatic cyclization using microbial transformations (Kim et al. 2019). Spectroscopic analysis confirmed that IX as such produced was a racemic mixture of (2S-) and (2R-) IX (Kim et al. 2019) and also in hop or hop pellets, IX and 8-PN were present as a racemate (Moriya et al. 2018). In vivo, IX is further enzymatically converted to 8-PN via hepatic P450 drug metabolizing enzymes and by gut bacteria (van Breemen et al. 2014). A strain of the anaerobic bacterium *Eubacterium limosum* (LMG P23546), a gut commensal in some individuals, has indeed been isolated before and used for efficient conversion of pure IX (> 99%) into 8-PN at up to 90% conversion efficacy (Possemiers et al. 2005). Martinez et al. (2015) studied enantiospecific pharmacokinetics of IX in rat and monitored the appearance of 8-PN (Martinez and Davies 2015). Yet, as (S‐) 8-PN was found excreted in the urine in greater amounts than (R‐) 8-PN, further enantiospecific in vivo bioactivity studies are needed.

**Fig. 1:**
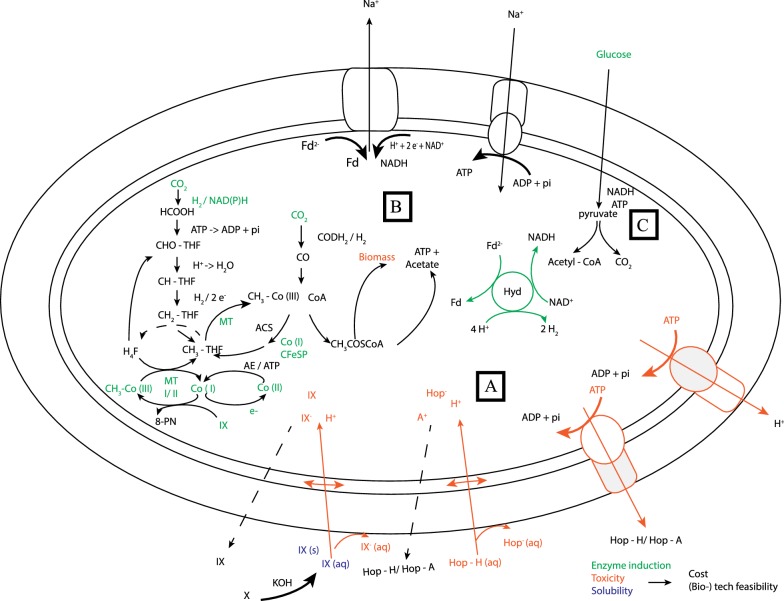
Schematic representation of presumed factors influencing the conversion of abundant hop metabolite xanthohumol (X) to 8-prenylnaringenin (8-PN). The process starts with conversion of X from spent hops into isoxanthohumol (IX) by chemical isomerization with KOH, followed by enzymatic demethylation of IX into 8-PN by *Eubacterium limosum*. Cellular transport of hop compounds may occur via diffusion or via specific transporters (A). Conversion of IX to 8-PN is suggested to occur via the Wood-Ljungdahl pathway in absence (B) or presence (C) of carbohydrates such as glucose. The major enzymes involved in *O*-demethylation of IX are methyltransferase I (MT I), corrinoid protein (CFeSP) with cobalt in the respective oxidation states Co (x), methyltransferase II (MT II), activating enzyme (AE), Coenzyme A (CoA), carbon monoxide dehydrogenase (CODH), acetyl-CoA synthase (ACS). Small adaptations were made from the schemes provided by Drake (1994), Sakamoto (2003), Studenik (2012), Jeong (2015). The green, red and blue parts in the figure may be important factors in the study of solubility of IX, toxicity of spent hops residuals and induction of conversion enzymes

Spent hops, a side product generated by industrial extraction of bitter acids from hop cones for the beer brewing industry, was presumed to be an economically interesting source of X and its isomer IX. Supercritical CO_2_ (sCO_2_) is the most common solvent for extraction of bitter acids from hop cones and has very high selectivity for the latter compounds. An interesting 8-PN bioproduction process could thus consist of alkaline pretreatment of X in spent hops (spent hop-X), yielding the isomerized spent hop-IX, and subsequent bioconversion of the latter into 8-PN. Traces of particular compounds have been quantified in spent hops (Krishna et al. 1986; Anioł et al. 2007; Aniol and Zolnierczyk 2008; Rój et al. 2015), yet, while the amount of X was almost not affected by sCO_2_ extraction, extensive characterization of the main other residuals in different batches of spent hops is not available. It is however clear that spent hops may still contain traces of a complex mixture of different compounds. Hop constituents, mainly the bitter acids, have next to their ‘hoppy taste’, a long-term use as preserving agent, minimizing bacterial spoilage. The molecules are highly active, requiring multiple bacterial resistance mechanisms to a heterogeneous mixture (Sakamoto and Konings 2003; Suzuki et al. 2006; Behr and Vogel 2009, 2010; Yin et al. 2018). Resistance includes both ionophore and oxidative stress induced mechanisms which made researchers in the early nineties conclude that any antimicrobial tested thus far could not simulate the stress induced by hop compounds in bacteria (Fernandez and Simpson 1993). To counteract the stress induced by the hop-derived ionophores, resistant cells, such as some beer spoilage lactic acid bacteria have used specific ABC or proton motive force dependent transporters to remove hop compounds or intercellularly released protons from the ionophores into the external environment (Sakamoto et al. 2001; Suzuki et al. 2002) (Fig. 1a). Based on the general acceptance of hop antibacterial effects and the low minimum inhibitory concentrations (MIC) reported by some researchers (Cermak et al. 2017; Bartmańska et al. 2018; Bocquet et al. 2019), it was anticipated that spent hop extracts would strongly affect the metabolism of *Eubacterium limosum*.

*Eubacterium limosum* can use the Wood-Ljungdahl (WL) pathway for demethylation of phenolic compounds (Genthner et al. 1981; Drake 1994; Liu and Suflita 1994). This pathway was thus suggested as the most likely metabolism for IX conversion (Fig. 1b) (Drake 1994; Studenik et al. 2012; Jeong et al. 2015). For methylotrophic reactions in *Eubacterium limosum*, inducible enzymes and complex patterns of diauxic growth and (co-) metabolic regulations have been described (Pacaud et al. 1985, 1986; Lindley et al. 1987; Cocaign et al. 1991; Loubiere and Lindley 1991; Loubiere et al. 1992; Lebloas et al. 1994, 1996). Whereas during growth on sugars, the WL pathway can be used to maintain the redox balance but there is no need that the pathway is coupled to energy conservation (Fig. 1c), in absence of sugars, the pathway must be coupled to net ATP synthesis such as via ion gradient-driven phosphorylation by ATP synthases (Müller 2003).

In this research, different strategies, physical (biomass entrapment), chemical (sCO_2_ and hexane (re-) extraction of (spent) hops), biotechnological (medium optimization, exploration of resting cell conversions) have been investigated in order to achieve fermentation of antibacterial industrial side stream spent hops. Major factors to optimize were solubility of IX, toxicity of spent hops residuals and induction of conversion enzymes (Fig. 1).

## Materials and methods

### Hops and spent hops treatments

Hop and spent hop pellets were obtained from hop manufacturing company Hopsteiner (Hopfen GmbH, Mainburg, Germany), Barth-Haas Group (Paddock Wood, Kent, United Kingdom) and from a local brewery (Van Steenberge, Ertvelde, Belgium). Spent hop pellets were isomerized under alkaline conditions and an IX rich precipitate was obtained by subsequent acid precipitation. In brief, 50 g of spent hops was suspended in 1 mol L^−1^ KOH. The suspension was stirred at room temperature during 90 min while the pH was kept at pH 13–14. Subsequently, the suspension was centrifuged 10 min at 3220 g (C5810, Eppendorf, Hamburg Germany). The pH of the supernatant was lowered to pH 5.6 using 10 mol L^−1^ HCl, leaving the suspension to precipitate overnight. The suspension was again centrifuged (10 min at 3220 g) and the pellet was washed three times using distilled water. Water from the pellet was removed with the use of a rotavapor (BÜCHI, Hendrik-Ido-Ambacht The Netherlands) and/or the pellet was directly suspended in ethanol or methanol. Alternatively, the precipitate was stored at − 20 °C until further use. Stock solutions in ethanol or methanol, standardized at 5 g IX L^−1^ were stored in the freezer at − 20 °C. IX used as positive control in the incubation studies was obtained via a dichloromethane/methanol (95/5, v/v) extraction of 35.6 g isomerized spent hop precipitate. The extract was purified with flash chromatography on silica gel (60–200 µm, ROCC): 12.4 g extract was dissolved in a minimal amount of ethyl acetate and this solution was eluted on 1500 g Silica using dichloromethane/methanol (95/5) as eluent. The fractions that contained IX as checked by TLC (Machery-Nagel, SIL-G25 (UV254) 0.25 mm) under 254 nm UV light were collected and evaporated in vacuo. This procedure was repeated three times, each time collecting the IX containing fractions. Finally, the obtained residue was triturated with diethyl ether and 800 mg final product (IX) was obtained. The purity was checked by comparison of the peak area with reference materials using HPLC–DAD (Hitachi Chromaster, VWR, Leuven Belgium). The mass of IX was confirmed by LC–MS (Agilent 1100 HPLC coupled to an Agilent G1956D MS detector with ESI ionization source, Diegem Belgium). The 1H-NMR spectrum was recorded on Brüker Avance 400 MHz NMR (Kontich Belgium) and found to be identical to that of the reference material (Possemiers 2007).

Hexane extraction of (spent) hops was done by batch extraction at room temperature based on the method described by Aniol and coworkers (Aniol and Zolnierczyk 2008). Supercritical CO_2_ (sCO_2_) extraction was performed on spent hops at lab scale as described by Van Opstaele and coworkers using Dionex SFE-703 supercritical fluid extractor (Dionex, Sunnyvale USA) (Van Opstaele et al. 2012). The sCO_2_ extraction was performed at the KAHO Sint-Lieven University College, Laboratory of Enzyme, Fermentation and Brewing Technology (Gent Belgium).

### Bacterial culture

The 8-PN-producing strain *Eubacterium limosum* LMG P23546, isolated by Possemiers et al. 2007 was used in all experiments (Possemiers 2007). Experiments were initially performed in brain heart infusion (BHI) medium (Oxoid, Hampshire UK) with addition of 0.5 g L^−1^ cysteine HCl (Merck, Darmstadt Germany) at 37 °C, using their methods. Bioconversion of pure IX (> 99%) into 8-PN by growing *E. limosum* in BHI medium at 25 mL scale was demonstrated by Possemiers and coworkers at a concentration of 25 mg IX L^−1^ and was used as positive control in this study (Possemiers et al. 2005). A minimal medium was previously optimized in our lab for maximal biomass production, starting from the medium reported by Leclerc et al. (1997) for isolation of acetogens from human colon (Leclerc et al. 1997) (data not shown). The composition consisted of glucose, 20 g L^−1^; (NH_4_)_2_SO_4_, 1 g L^−1^, NaHCO_3_, 1.24 g L^−1^; MnCl_2_(H_2_O)_4_, 0.55 g L^−1^; MgSO_4_.7H_2_0, 0.1 g L^−1^; CaCl_2_, 0.02 g L^−1^; yeast extract, 1.5–5 g L^−1^, cysteine, 0.5 g L^−1^. The minimal medium was used as basis for further supplementation in this research. Additional products used for medium optimization were reinforced clostridial medium (RCM) (Oxoid, Hampshire UK), yeast extract, methanol, anaerobic vitamin and trace mix (Balch et al. 1979; Pacaud et al. 1985; Greening and Leedle 1989). The initial pH of all media was around 6.8–7.2. Biomass entrapment was done using zeolites Zeowater (2.5–5 nm) (Zeocem, Bystré Slovak republic). Resazurin was added as a redox indicator in all experiments. All media were autoclaved at 121 °C for 15 min. Subsequent manipulations were done in the anaerobic chamber with a headspace composition H_2_/CO_2_/N_2_ of 10/10/80 (v/v/v) (Airliquide, Belgium). The fermentation volume was 25 mL and incubations were done using 100 mL vessels, sealed with rubber stoppers. Cultures were incubated at 37 °C and were exposed to the atmosphere of the anaerobic chamber via a sterile needle and filter. At given time points, samples were collected using syringes. Inocula were preserved at 4 °C as freeze-dried pellets (LyoBeta Mini, Telstar) of 10 mL of 24 h grown cultures in BHI medium. Culture experiments with growing *E. limosum* were performed by inoculation of 100 μL of 24 h revived culture in 25 mL of fresh medium and IX or spent hop-IX (5 g IX L^−1^) was added concomitantly. Resting cell suspensions (concentrated non-growing metabolically active cells, cNGMA) were obtained by centrifugation of stationary phase biomass (non-growing metabolically active cells, NGMA) for 10 min at 4500 g (C5810, Eppendorf, Hamburg Germany). Conversion was expressed as molar relative conversion of IX (fraction at the start versus at the end of the experiment), specific conversion was calculated using the molar relative conversion of IX per unit of biomass, the IX concentration in the extract and the spiked volume of extract. Basic metabolism was monitored by short chain fatty acid (SCFA) production. SCFA were extracted from the samples with diethyl ether and determined with a Di200 gas chromatograph (GC-FID) (Shimadzu,’s-Hertogenbosch The Netherlands) (Nollet et al. 1997). Bacterial growth was analysed as an increase in optical density at 600 nm. Analysis was done with use of spectrophotometer DR 3900 (Hach Lange, Belgium). Dilutions were made in physiological water (0.85% NaCl) in case the OD exceeded 0.4. The biomass cell wet weight content was measured as the weight of the biomass pellet after samples were centrifuged at 12,500 g for 10 min. Cell Dry Weight (CDW) was measured after the water content was evaporated at 105 °C for 24 h.

### Analysis

Extractions and analysis of X, IX and 8-PN were based on the work of Possemiers et al. 2005 and Bolca et al. 2007 (Possemiers et al. 2005; Bolca et al. 2007). Quantification was done by HPLC (Hitachi Chromaster, VWR Leuven, Belgium) equipped with a Photodiode Array Detector (Hitachi Chromaster 5430). A C18 reversed-phase column (Kinetex, 250 × 4.6 mm, 5 μm) was used in combination with a gradient composed of solvent A (water acidified with 0.025% (v/v) formic acid) and solvent B (methanol acidified with 0.025% (v/v) formic acid). The gradient was: 0–12.8 min: 45% B in A; 12.8–17.9 min: 95% B in A and 17.9–21.8: 45% B in A. The flow-rate was 1 mL min^−1^ and the column temperature 35 °C. Detection was done simultaneously at 295 nm (for IX, 8-PN) and at 370 nm (for X) using diode array detection. Peaks were identified by comparison of the retention times with those of authentic isolated reference compounds (Possemiers 2007), as well as by inspection of the respective UV spectra. External 4-point calibration curves were established for the compounds X, IX, 8-PN together, in a solution of 5 g L^−1^ in methanol (R^2^ > 0.99). Concentrations were calculated based on peak area integration. For solid samples, X, (IX, 8-PN) were extracted with methanol (Nikolic and Van Breemen 2012). The samples were sonicated (Bandelin Sonorex) for 1 hour and kept in the fridge (4 °C) for 3 h. The upper phase after centrifugation for 15 min at 1300 g was analysed. For fermentation samples, 1 mL was mixed with 1 mL sodium acetate buffer (0.1 mol L^−1^, pH 5) before extraction. The diluted samples were subsequently extracted twice with 5 mL diethyl ether. The solvent was evaporated at room temperature under a N_2_ stream and the residue was dissolved in methanol, transferred into HPLC vials and stored at − 20 °C prior to analysis. As an internal standard 5 g L^−1^ 4-hydroxybenzophenone in methanol was used.

## Results

### Xanthohumol content in hop and spent hop samples

The xanthohumol (X) content differed considerably depending on the hop variety (Table 1) and this was presumed to be also the case for other residual compounds (not quantified). The IX content of the precipitates obtained after spent hops pretreatment was in the range of 5–10 m% on wet weight.

**Table 1:** Quantification of xanthohumol in diverse hop and spent hop samples, as determined by HPLC–DAD

Hop variety	Xanthohumol (X) (m%)^a^
Herkules	
Hop pellets	1.00 ± 0.01
Spent hop pellets	1.45 ± 0.01
Golding	
Hop pellets	0.22 ± 0.01
Spent hop pellets	0.36 ± 0.03
Blind sample	
Hop pellets	1.22 ± 0.10
Spent hop pellets	1.39 ± 0.07
Herkules	
Spent hop pellets	1.22 ± 0.07
Hallertauer exp. variety	
Spent hop pellets	2.14 ± 0.03
Hallertauer magnum	
Spent hop pellets	0.64 ± 00
Summit	
Hop pellets	1.08 ± 0.03

^a^Concentrations are expressed as mass percentage on the samples as such (without consideration of dry weights)

### IX conversion in food and feed grade bioconversion medium

The rich brain heart infusion (BHI) medium was used to deliver the proof of concept for conversion of pure IX at 25 mg IX L^−1^ by *Eubacterium limosum* (Possemiers 2007). Yet, the high cost and presence of material from animal origin made this medium inappropriate for industrial production or nutritional applications. Initial efforts to look for a suitable alternative medium focused on the design and stepwise supplementation of a minimal medium, using different 5-day static incubations (Table 2). Vitamin and trace metal mix for anaerobes and/or yeast extract were added to account for sufficient enzyme and cofactor production (Balch et al. 1979; Greening and Leedle 1989). The Wood-Ljungdahl substrate methanol (up to 2% v/v) was added for increased solubility of IX in combination with biomass entrapment with zeolites (5% w/v), and manganese was omitted specifically as it has been described to enhance the detrimental effects of hop ionophores (Behr and Vogel 2009, 2010). In order to observe possible (co-) metabolic interactions, glucose was omitted or decreased to the same amount as present in BHI medium (2 g L^−1^), ethanol was used as alternative for methanol to supply IX and the headspace composition was altered between N_2_ versus H_2_/CO_2_/N_2_ of 10/10/80 (v/v/v). Using a minimal medium, efforts needed to maintain sufficient anaerobic conditions upon addition of IX were cumbersome and moderately reproducible (colour change of redox-indicator resazurin). Increasing the concentration of reducing agent may have led to accumulation of toxic oxidized intermediates as no improvements were obtained. Using a different approach, the commercially available complex medium reinforced clostridial medium (RCM), which can be produced in a food and feed grade fashion, was included in the study. Although some conditions using a minimal medium enabled growth, overall the results were not satisfying with regard to conversion (Table 2).

**Table 2:** Effect of different types of medium on growth and conversion of pure IX at 25 mg IX L^−1^ by *Eubacterium limosum*

Condition (10 H_2_/10 CO_2_/80 N_2_ (v/v/v))	Growth (OD_600_)	Molar relative IX conversion (HPLC–DAD)
BHI (reference)	++	++
MM	+	–
MM ± additions	+	–
MM, omission of glucose	–	–
MM 2 g L^−1^ glucose	–	–
MM 10 g L^−1^ glucose	+	–
BHI + zeolite (5% w/v)	++	++
MM + zeolite (5% w/v)	++	+
RCM	++	++

Scoring was made relative to the incubation in BHI as positive control (‘++’: 75–100% of positive control (reference); ‘+’: 50–75% of positive control; ‘-’: < 50% or absence). 100% growth in the case of the reference corresponded with a 20-fold increase in biomass (expressed as cell dry weight) compared to the inoculum

*BHI* brain heart infusion medium, *MM* minimal medium, *RCM* reinforced clostridial medium

Trace elements for anaerobes could not increase conversion in minimal medium, suggesting that (micro-) nutrient requirements were not the major factor for low IX conversion in the minimal medium. Lowering the glucose levels in the minimal medium led to poor growth and metabolism. A combination of solvent addition for increased solubility with zeolite entrapment of the biomass initially led to increased biomass growth and short chain fatty acid production and was the only way for conversion in minimal medium in the same range as with BHI medium. Large aggregates of biomass were formed with the zeolites and butyrate and acetate production were 2-3 fold higher as compared to absence of zeolites. The latter production of basic metabolites resembled to values obtained for incubations in absence of IX source (data not included). The composition of RCM was finally selected as food and feed grade equivalent to BHI medium in terms of growth and conversion of 25 mg IX L^−1^ to up to 30 mg IX L^−1^. A controlled headspace (pressure and composition) was beneficial compared to a non-controlled confined headspace with regard to reproducible conversion. There was no difference between use of methanol (established methylated substrate for *E. limosum*) versus ethanol to supplement IX with regard to growth and conversion.

In search for the alternative production medium, rich substrate and/or biomass entrapment with zeolites were the most important factors.

### The bacteriostatic effects of isomerized spent hop extract

Although a suitable production medium for pure IX conversion could be identified, any conversion of IX in isomerized spent hop extract (spent hop-IX) failed, confirming the traces of diverse hop compounds in spent hop extracts were antibacterial to *E. limosum*. A presumed technical feasible strategy to improve fermentation consisted of a supplemental spent hop pretreatment step, additionally removing the antibacterial residuals. As extra pretreatment of the spent hops, lab scale supercritical CO_2_ and hexane extraction were performed on different batches of (spent) hops, including differences in varieties, drying and storage conditions. Although there was no growth in any liquid incubation with spent hop-IX, growth was observed after a lag phase upon subsequent transfers into fresh medium (1–5% inoculation), whether or not supplemented with pure IX. The latter observation suggested very low minimum inhibitory concentrations (MIC) for spent hop compounds and/or a bacteriostatic yet not bactericidal effect of spent hops on *E. limosum*. Additional pretreatments and/or re-extractions on the side stream spent hops could not eliminate the inhibitory effects of the complex matrix of spent hop extract in the liquid incubations.

### Exploration of the non-growth phase: stationary phase conversion of IX

**Fig. 2:**
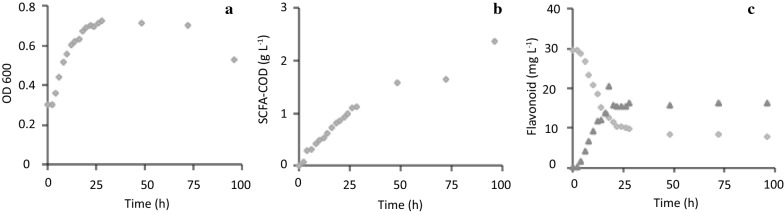
Course of growth (**a**), basic metabolism, i.e., production of acetate and butyrate summed as chemical oxygen demand (COD) (**b**), and conversion of pure IX at 30 mg IX L^−1^ (rhombi: IX; triangles 8-PN) (**c**) during growth of *Eubacterium limosum* in reinforced clostridial medium at 37 °C

**Fig. 3:**
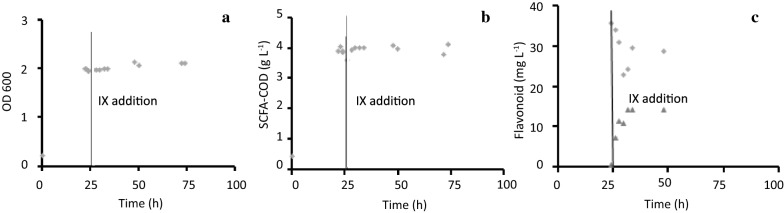
Course of growth (**a**), basic metabolism, i.e., production of acetate and butyrate summed as chemical oxygen demand (COD) (**b**), and conversion of pure IX at 30 mg IX L^−1^ (rhombi: IX; triangles 8-PN) (**c**) during stationary growth of *Eubacterium limosum* in reinforced clostridial medium at 37 °C

The presence of diverse hop compounds in spent hop extract strongly impeded bacterial growth, until very low levels of around 1–5 mg IX L^−1^ medium. Therefore, the relation between IX conversion, growth and basic metabolism (acetate, butyrate production) was studied in more detail in growth and non-growth conditions (Figs. 2 and 3). In the growing cells experiment (i.e., IX was dosed at 30 mg IX L^−1^ concomitant with inoculation of 0.5% of *E. limosum*), the culture started to grow immediately, and showed exponential growth till around 20 h, reaching an optical density (OD) of about 0.8 (Fig. 2a). Short chain fatty acids (SCFA) butyrate and acetate, summed as chemical oxygen demand (COD), occurred during the exponential phase at a constant rate and slowed down upon reaching the stationary phase (Fig. 2b). The pH after 20 h was 6.1. The rate of SCFA production was elevated at the end, possibly due to cryptic growth and further lowered the pH to 5.6. IX was converted into 8-PN during the exponential phase, but no further conversion occurred upon prolonged incubation (Fig. 2c). IX conversion to 8-PN stopped at a relative molar conversion of around 65% and the stop coincided exactly with the onset of the stationary phase.

In the stationary phase experiment (i.e., IX was dosed at 30 mg IX L^−1^ when the culture had reached the stationary growth phase), arrest of growth was confirmed through successive OD determinations at close time points, indicating a stable and high OD value of 2 as compared to 0.8 when IX was dosed from the start (Fig. 3a). Stable SCFA concentrations were measured during the stationary phase and concentrations were more than double the amount as compared to incubations with IX present from the start in the growing cell experiment (Fig. 3b). The high SCFA accumulation resulted in a pH drop to pH 5 after 20 h of incubation. Although the relative molar conversion only reached around 53%, the initial conversion rate was much higher than for growing cells, yet, homogeneous sampling of 8-PN and IX was hampered in presence of the dense biomass (Fig. 3c). Cells which were able to convert IX into 8-PN in the stationary phase, represent non-growing metabolically active (NGMA) cells. There was almost no lag phase observed for conversion, the CO_2_ and H_2_ provided in the headspace of the incubation vessels may have already induced the WL enzymes during the growth phase.

Due to the altered consistency of the NGMA cells, the initial IX level appeared somewhat higher, yet the same volume of IX solution was spiked as for the growing experiment. Conversion values between 16 and 24 h were further confirmed in independent optimization experiments in time with coefficients of variation below 11% (data not shown).

Based on the growth experiment (Fig. 2), reasons for the cessation of IX conversion at the transition of the exponential phase to the stationary phase were initially thought to be: (I), conversion is growth-related and cannot occur during non-growth, (II), the pH has dropped too much in the batch incubation to allow further metabolism (pH 6.1), (III), toxicity of the end product has ceased conversion. The subsequent results of the stationary phase experiment (Fig. 3) demonstrated that: assumption (I) is not valid, i.e., IX conversion was not restricted to growth of the culture and yet presence of a lot of biomass was beneficial for rapid conversion and (II), pH effects may have occurred more rapidly because the pH at the moment of IX addition was already low (pH 5).

### Spent hop-IX conversion: sensitivity for pH and growth phase

In order to further evaluate the effects of growth phase and pH on IX conversion, conversion of IX by growing and stationary phase cells was compared at increasing pH values. Above pH values of 7.5–7.7, lag phases of more than 10 h occurred for growing cells upon inoculation. Yet, higher pH values allowed to further increase the concentration of IX to 35 mg L^−1^ as a consequence of improved solubility.

**Fig. 4:**
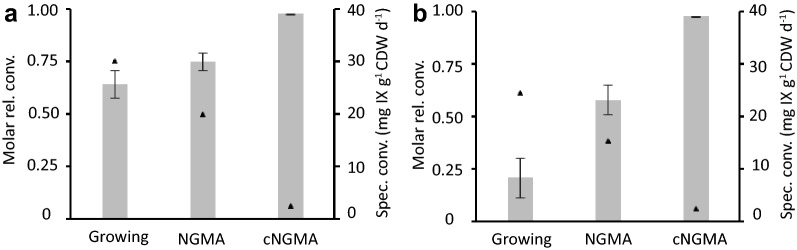
Conversion of 35 mg IX L^−1^ by *Eubacterium limosum* at the maximal pH for growth of 7.5–7.7 for IX (n = 3) (**a**) and three different sources of spent hop-IX (**b**). Bars indicate molar relative conversion, triangles indicate specific conversion for the growing biomass, non-growing metabolically active (NGMA) cells and 10× concentrated NGMA (cNGMA)

When the pH of the stationary phase biomass (non-growing metabolically active cells, NGMA cells) was kept at 7.5–7.7, fair and robust conversion of 35 mg IX L^−1^ occurred, irrespective of IX source (pure IX versus 3 different batches of spent hop-IX) (Fig. 4). Conversion was 100% in case stationary phase biomass suspensions were used (i.e., concentrated stationary phase cells or cNGMA, obtained by centrifugation). Although high pH was beneficial for spent hop-IX conversion, the elevated pH on the other hand lead to impaired growth in growing cells experiments, lowering also pure IX conversion in this case.

## Discussion

In this research, the aim was to develop a fermentation strategy for bacterial conversion of isoxanthohumol (IX) into the potent phytoestrogen 8-prenylnaringenin (8-PN), using antibacterial spent hops as source of IX. IX conversion is an *O*-demethylation reaction and was therefore suggested to be part of the Wood-Ljungdahl (WL) pathway of *Eubacterium limosum* (Genthner et al. 1981; Drake 1994; Liu and Suflita 1994). Different parameters such as growth phase, pH, headspace and medium composition needed to be optimized in order to achieve maximal solubility and enzyme induction next to low redox and toxicity. A commercial complex rich medium, reinforced clostridial medium (RCM) and controlled headspace, were selected as most efficient with regard to medium requirements. Diauxic growth patterns were not observed, on the contrary, the demethylation of IX into 8-PN was enhanced in presence of glucose and seemed unaffected by use of the WL substrate methanol as solvent. Improved IX conversion in complex rich medium was mainly attributed to improved solubility of IX, pH stability, low and stable redox and protective effects of lipid materials and proteins. Stevens et al. previously demonstrated that carbohydrates considerably influenced the solubility of IX by forming soluble complexes (Stevens et al. 1999a). The (apparent) solubility of IX in either water, 5% ethanol, beer at 1% w/w carbohydrates and different brewing worths at 8.4 and 13% w/w of carbohydrates was respectively 5, 6, 27, 40 and 43 mg IX L^−1^ at 23 °C. Next to carbohydrate complexation reactions, IX also binds to proteins (Stevens et al. 1999a).

Although initially growth and conversion seemed related (IX conversion was only observed concomitant with considerable biomass formation), hourly sampling during growing and resting cell experiments revealed that stationary phase cells may well be used for IX conversion. The latter observation was of great interest, given the fact that the most interesting economic source of IX, spent hops, acted as a strong bacteriostatic agent. Following this line, spent hop-IX could indeed be converted using stationary phase biomass. Stable low redox and/or energy levels may have been more optimal in the densely grown culture, providing cellular resistance to ionophore and oxidative stresses. Although growth was increasingly impaired at pH values above pH 7.5, conversion with non-growing biomass was slightly further improved after increasing the pH during conversion to 7.8–8 (results not shown). pH values may affect bacterial cultures via different ways, including altered membrane permeability, changes in cellular physiology and/or expression of transporters (Dilworth et al. 1999; Petrackova et al. 2010; Siliakus et al. 2017).

**Fig. 5:**
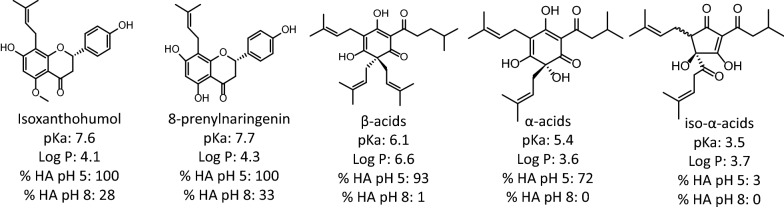
Theoretic calculations of undissociated fractions (HA) of substrate IX, product 8-PN and traces of bitter acids in spent hops at pH 5 (pH of stationary phase cultures in absence of pH control) versus pH 8 (maximal tolerable pH value for *Eubacterium limosum*), based on reported pKa values and the Henderson–Hasselbalch equation (Simpson and Smith 1992). Considering the relatively high (estimated) log P values of the most abundant antibacterial hop compounds, bioavailability via diffusion into the bacterial cells may be much altered based on the pH of the medium. %HA: undissociated fraction of the weak acids. The structures in the figure are the most commonly represented stereo form of the compounds

The enzymes of the WL pathway are not membrane bound (Hess et al. 2013) which requires effective transfer of the substrates into the cell via passive diffusion or transporters. The shift in pH to higher values may increase the dissociation of the protic weak acids (bitter acids, IX and 8-PN) and therefore affect the diffusion rates. In addition, also the aqueous solubility at room temperature of 8-PN almost triples for an increase in pH from neutral to pH 8 (from 3 to 8 mg L^−1^) (Riis et al. 2007). The theoretic dissociated fraction of the weak acids was approached using the Henderson-Hasselbalch equation for a range of pKa values for bitter acids, IX and 8-PN (Verzele and De Keukeleire 1991; Simpson and Smith 1992; Hermans-Lokkerbol et al. 1997; Irwin and Shoichet 2005; Cattoor 2012; NCBI 2019; PhytoHub 2019a, 2019b; PubChem 2019) and indeed confirmed pH 7.8–8 could be the most optimal value for fermentation (Fig. 5). For the bitter acids, the potential pH dependent differences in bioavailability may be most interesting, given their lower range of pKa values and yet fairly lipophilic (estimated) log P values of around 3.9–6.2. At pH 8, the bioavailability via diffusion of a considerable fraction of bitter acids may be much decreased (the undissociated fraction is close to zero) whereas a large fraction of IX and 8-PN may remain available for diffusion into the cells.

Finally, the whole process would intuitively benefit from enzymatic conversion as compared to whole cells, overcoming energy spilling reactions by ionophores and solubility issues. Demethylation of IX into 8-PN seems a one-step reaction, yet, the key enzymes catalysing the methyl cleavage are the *O*-demethylases, consisting of a four-component cytosolic enzyme system (Studenik et al. 2012) (Fig. 1b). Specifically, methyltransferase I (MT I) binds the substrate, catalyses the cleavage of the C–O bond and the transfer of the methyl group to a copper-corrinoid protein ([CO^I^]-CP), yielding demethylated products and [CH_3_-CO^III^]-CP. Subsequently, methyltransferase II (MT II) mediates the transfer of the methyl group from [CH_3_–CO^III^]-CP to tetrahydrofolate (FH4), producing methyl-FH4 and [CO^I^]-CP. The enzyme system requires low redox, which is mediated by activating enzymes (AE). In order for the reactions to occur, the redox has to be below − 290 mV versus the standard hydrogen electrode (SHE) at pH 7.5 in presence of AE and ATP or below − 450 mV in their absence (Studenik et al. 2012). The genes encoding MT-I, CP and MT-II are organized in one transcription unit as an operon, while the AE gene is located separately (Studenik et al. 2012). Chen and coworkers identified, cloned and expressed the *0*-demethylase system of intestinal *Eubacterium limosum* ZL-II in *Escherichia coli* (Chen et al. 2016). Enzymatic demethylation of test substrate secoisolariciresinol (SECO) was confirmed using a reconstruction of the complete *O*-demethylase reaction system in vitro but the reaction efficiency was weak (about 6% of SECO was converted after reacting for 8 h). Since the *O*-demethylase activity requires a cooperation of four different proteins, it may be challenging to reach high efficiency using in vitro. So far, the highest bio-conversion capacity of IX from economic sources into 8-PN was obtained in this research, using whole non-growing metabolically active cells of *Eubacterium limosum*. The boundaries for production set at lab scale from this study are being used for optimization to a scalable bioprocess.

## Data Availability

Data is available on request.

## Abbreviations

8-PN: 8-prenylnaringenin
X: Xanthohumol
IX: Isoxanthohumol
sCO_2_: Supercritical CO_2_
MIC: Minimum inhibitory concentration
WL: Wood-Ljungdahl
NGMA: Non-growing metabolically active
cNGMA: Concentrated non-growing metabolically active
BHI: Brain heart infusion
RCM: Reinforced clostridial medium
MM: Minimal medium
